# Investigation on application of filling mining technology pass through abandoned roadway of reclaimed working face

**DOI:** 10.1371/journal.pone.0291519

**Published:** 2023-09-29

**Authors:** Zi-jie Hong, Shun Chen, Zhen-hua Li, Zhi-xuan Chen, Xu Cui, Lei Xu

**Affiliations:** 1 School of Civil Engineering, Henan Polytechnic University, Jiaozuo, 454003, China; 2 The project of Henan Key Laboratory of Underground Engineering and Disaster Prevention (Henan Polytechnic University), Jiaozuo, China; University of Science and Technology Beijing, CHINA

## Abstract

This paper aims to analyze the roof stability when the reclaimed working face passes through abandoned roadway. The mechanical model of main roof in abandoned roadway was established for the purpose of theoretical analyses. To ensure the stability of the abandoned roadway, the strength formula of backfill body was deduced. The optimum ratios among different compositions of the filling material were determined by experiment, while the viscosity, bleeding, hydration temperature and compressive strength of filling material were also studied. Test results indicated that the optimum ratio among coal ash, lime and compound activator is 80:15:5, and the ideal water cement ratio is 0.7:1. It was also found that no bleeding occurred, the rheological behavior of slurry presented shear thinning fluid and the hydration temperature of filling body was relatively stable which is mainly maintained at 40°C. The uniaxial compressive strength of filling material with 28 and 90 days curing were 3.35 and 6.62 MPa respectively. Under a confining pressure environment, the filling material presented an obviously plastic deformation. Field test showed that the filling rate was almost 100%, when working face passed through abandoned roadway, the surface of filled body was complete and no roof collapse was triggered. Therefore, a better bonding effect was proved for the filling body.

## Introduction

Because of the backstopping coal mining approaches in China are quite undeveloped, such as roadway mining, disordered mining, bord and pillar mining and excessive contact lanes, all wasted too much coal resource and resulted in low recovery rate is caused [1,2]. The method of increasing the recovery rate by rearranging the working face is called “reclaimed technique” [3]. The reclaimed working face makes the above-mentioned development roadway or preparation roadway become waste roadway [4,5]. Therefore, it is necessary to deal with the problem of passing through abandoned roadway under the advancing influence of reclaimed face. Meanwhile, due to the influence of the advancing pressure on working face, the stability of the roof is reduced [6–9]. When the working face passes through the abandoned roadway, both roof settlement and collapse occurred which threaten the safety production of the working face [10,11].

To deal with the deformation and collapse issues in the abandoned roadways, some traditional methods have been proposed. Which includes the dense pillars or wooden planks support [12–14], the bolt and anchor cable reinforcement, grouting reinforcement combined with anchor and paste material filling [15,16]. In order to study the reinforcement and supporting technologies in the abandoned roadway applications, various researches have been conducted. Wu et al [17] established the filling mechanics model and proposed the wood slag filling technology. Zhao et al. [18] studied the application of fly ash in traditional paste fillers and obtained different properties of paste fillers. Ma et al [19,20] studied the backfilling method of near horizontal filling, and adopted high moisture content materials to realize the mining method of water-preserving coal resources. Yılmaz et al [21] used the waste lane filling method to keep the surrounding rock in the initial stress state and improve the bearing capacity. Regard to the disadvantages, wooden support provides a poor stability in abandoned roadway, and chemical grouting is expensive, poisoning and corrosive. Grouting reinforcement combined with anchor can create a better roof stability in abandoned roadway, but it is still quite difficult to effectively control the plastic deformation and failure in abandoned roadway [22]. By comparison, filling method in abandoned roadway ensures that the surrounding strata is in the initial stress state and it increases the carrying capacity [23].

Filling mining is an important green mining technology and effective method to solve difficult problems [24,25]. At the same time, solid backfill, paste backfill and high water material backfill technology has achieved good results in coal mines in China [26–28]. Therefore, filling method has been rapidly developed, but still in the exploratory stage in terms of the filling material compositions and its strength. The initial investment required for backfilling equipment and large system scale, high cost of filling materials, and low filling strength cannot guarantee the smooth through abandoned roadway.

In order to ascertain the key problem when reclaimed working face passing through abandoned roadway and study how to maintain an efficient mining of the reclaimed working face, Shanxi Coal Industry has been selected as the engineering background. Mechanical analysis, laboratory experiments and industrial test were all conducted to study the filling strength and the proportion of filling material. Meanwhile, field work had achieved good results and ensured working face safe mining.

## Discussion on the filling strength

The filling method used in abandoned roadway utilizes the filling material to increase the bearing capacity of surround strata so that a good mining effect can be achieved. Therefore, the strength of filling body is the most important parameter. In order to ensure the stability of the abandoned roadway, the strength parameter was studied by theoretical analysis.

**Fig 1:**
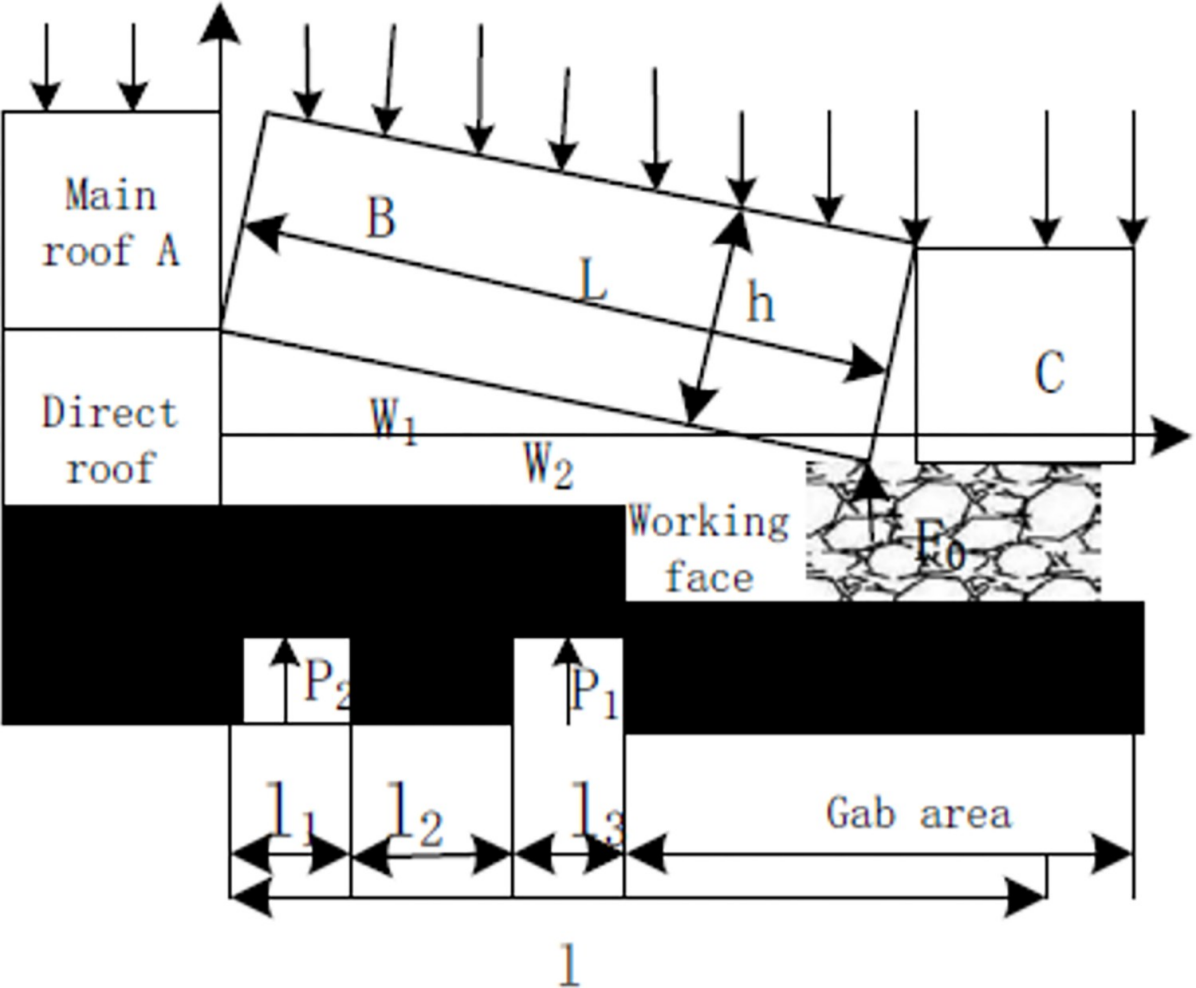
Mechanical model of key block.

After the slurry is filled with abandoned roadway, the crushed rock and filling body are cemented together to form a complete structure. When the abandoned roadway is influenced by mining activities, the filling body is compressed to support the roof, hence the possible large subsidence is prevented. It is because that the cemented body becomes to a part of the immediate roof which inhabits the large roof caving and collapse. The influence of advance mining on the roof stress state determines the strength of backfill body, therefore, the mechanical model was established based on the mechanical state of the roof as shown in Fig 1.

**Fig 2:**
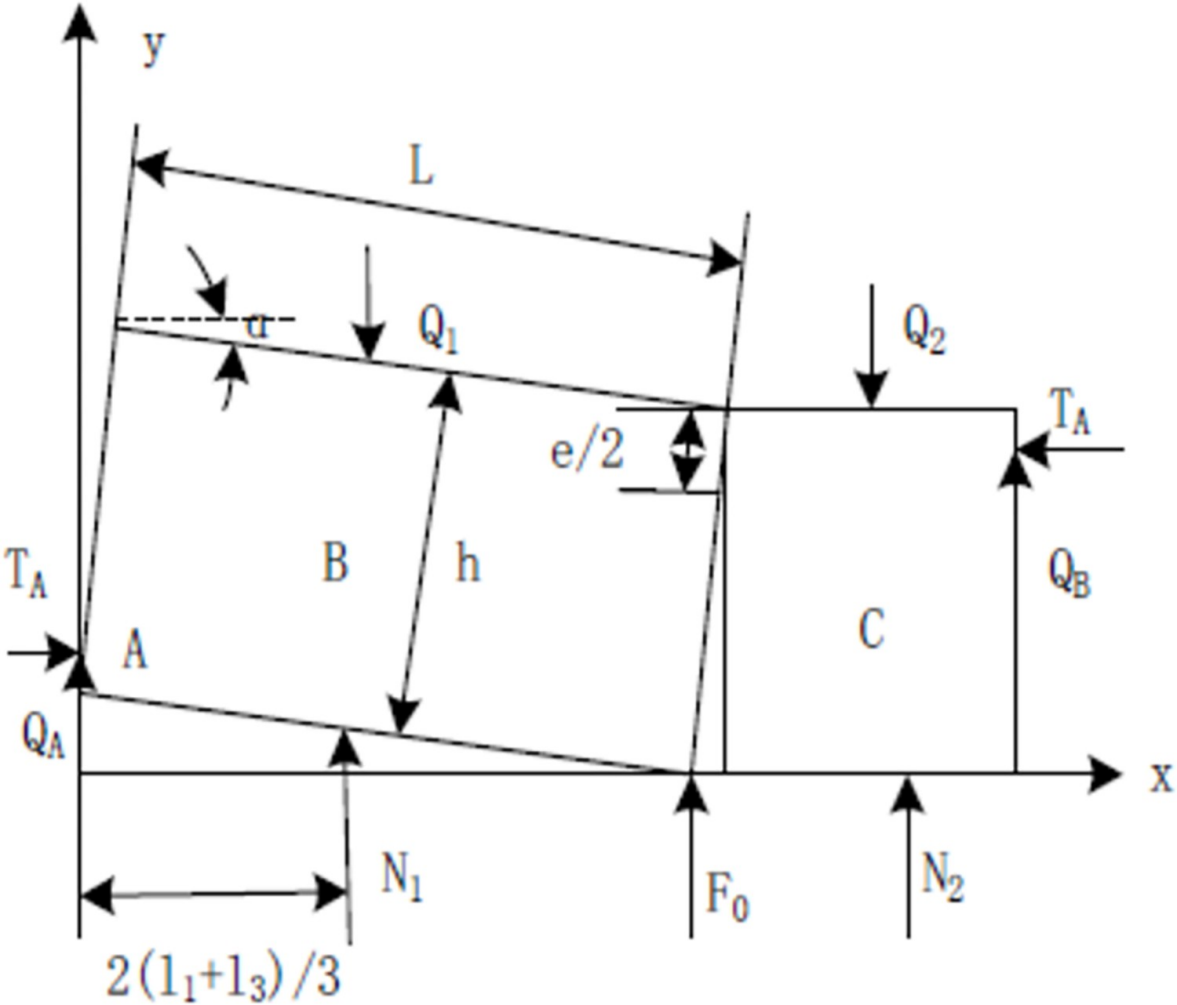
Analysis of key block mechanics.

When working face reached to abandoned roadway, it is most affected by mining activities, and the mechanical analysis of key block is shown in Fig 2. Therefore, the force analysis is carried out when l_2_ is 0. The force balance of blocks B and C is obtained, the equation is listed as follows:

$$\frac{2(l_{1}+l_{3})N_{1}}{3}+F_{0}L\cos\alpha +T_{A}\left(h-e-\lambda \right)-\frac{Q_{1}L\cos\alpha }{2}=0$$ (1)

Where Ɩ_1_ is the width of abandoned roadway, Ɩ_2_ is the width of coal, Ɩ_3_ is the second width of abandoned roadway, N_1_ is the direct support force for the key block B, α is the turning angle of B deviation to the gab area; T_A_ is the horizontal thrust of B and C; Q_1_ is B load and weight, F_0_ is the supporting force of gangue for B; e is the distance between the B and C blocks; L is the fracture distance of B along the advancing direction, λ is the rotation subsidence value of B.

For the force analysis of block B, the Eq (2) can be obtained:

$$Q_{1}=Q_{A}+F_{0}+N_{1}$$ (2)

Where Q_A_ is the shear stress of B.


μ=(h−e−λ) (3)

Eqs (2) and (3) are substituted into Eq (1), hence Eq (4) is obtained:

$$T_{A}=\frac{1}{\mu }(\frac{Q_{1}L\cos\alpha }{2}-F_{0}L\cos\alpha -\frac{2(l_{1}+l_{3})N_{1}}{3})$$ (4)

$$Q_{A}=Q_{1}-F_{0}-N_{1}$$ (5)

When B does not slip at A point, it must meet the following critical conditions [29]:

$$T_{A}.tan\varphi =Q_{A}$$ (6)

From the Eqs (4)–(6) the following results are obtained:

$$N_{1}=\frac{6\mu (Q_{1}-F_{0})+(6F_{0}-3Q_{1})L\cos\alpha tan\varphi }{6\mu -4(l_{1}+l_{3}).tan\varphi }$$ (7)

e is the distance between the B and C blocks [29], it can be calculated according to Eq (8):

$$e=\frac{h-L\sin\alpha }{2}$$ (8)

F_0_ is the supporting force of gangue for B, it can be calculated [30] according to Eq (9):

$$F_{0}=\int_{l}^{L\cos\alpha }d_{f}L_{1}k_{m}dx$$ (9)

Where:λ=Lsinα

$$Q_{1}=bL(h\gamma_{1}+q)$$

Stress balance analysis of block 3, the Eq (10) can be achieved: where the P_1_ is located at Ɩ_3_/3, P_2_ is located at Ɩ_1_/2.


$$\left\{ \begin{array}{l} \frac{P_{2}l_{1}}{2}+P_{1}(l_{1}+\frac{l_{3}}{3})+F_{0}L\cos\alpha -\frac{2(W+N_{1})(l_{1}+l_{3})}{3}=0\\ P_{2}+P_{1}+F_{0}=W+N_{1} \end{array}\right.$$ (10)

According to the Eq (10), the following equations are obtained:

$$P_{1}=\frac{l_{1}+4l_{3}}{3l_{1}+2l_{3}}.(W+N_{1})-3F_{0}\left(2L\cos\alpha -l_{1}\right)$$ (11)

$$W=W_{1}+W_{2}=b(l_{1}+l_{3})(D\gamma_{0}+h_{t}\gamma_{1})$$ (12)

Where P_1_ is working resistance of support, in kN; D is the seam thickness in m; h_t_ is the height of immediate roof in m; b is the bracket width in m; γ_1_ is the average bulk density of rock strata in kN/m^3^; W_1_ is the direct roof load; W_2_ is the top coal load; P_2_ is the resistance of roadway roof support in kN; γ_0_ is the bulk density of coal in kN/m^3^.

L is the fracture distance of B along the advancing direction [31], which can be calculated from Eq (13):

$$L=h\sqrt{\frac{R_{T}}{3q}}$$ (13)

Where h is the thickness of B; q is the load carrying capacity of B in MPa; R_T_ is the main roof tensile strength in MPa.

L_1_ is the lateral fracture distance of B [31], which can be calculated from Eq (14):

$$L_{1}=L[\sqrt{\frac{1.3L^{2}}{X^{2}}+102}-\frac{10L}{X}]$$ (14)

Where X is working face length in m.

The compressive amount of caved gangue d_f_ is calculated by Eq (15):

$$d_{f}=x\tan\alpha +\frac{\gamma_{1}H}{k_{g}}-[D-D(1-\eta )k_{d}-h_{t}(k^{'}-1)]$$ (15)

Where H is the overlying strata height of immediate roof in m; η is the coal mining rate; k_d_ is the coal expansion factor; k is the expansion coefficient of immediate roof.

Filling body supporting strength can be obtained from the above analysis:

$$\sigma =\frac{\left\{\left[N_{1}+(l_{1}+l_{3})\gamma_{1}h_{t}L_{1}-\frac{\sigma_{t}h_{t}^{2}}{3}](l_{1}+l_{3}\right)\}\frac{1}{f_{t}}-P_{1}(2l_{1}+l_{3}\right)}{l_{1}^{2}L_{1}}$$ (16)

Where k_m_ is the gangue support coefficient; the friction coefficient of tanφ is 0.2; f_t_ is the concentration factor, according to the empirical formula, the value is 2.18; σ_t_ is the tensile strength of immediate roof in MPa.

According to the mine geological data, the parameters are selected as follows: b = 1.5 m, γ_1_ = 25 kN/m^3^, h = 4m, q = 0.196 MPa, k_m_ = 5 MPa/m, X = 115m, R_T_ = 3.52 MPa, D = 5.5m, γ_0_ = 13 kN/m^3^, H = 180 m, η = 0.8, k_d_ = 1.5, k^,^ = 1.5, h_t_ = 4.2 m, σ_t_ = 4.56 MPa, k_g_ = 1000 MPa/m; α = 10°. The parameters are substituted into Eqs (7), (11) and (16), it is concluded that the strength of the supporting body is 3.3 MPa.

## Research and discussion on filling material

### Experimental raw materials

#### Fly ash

The fly ash used in the experiment is produced by Yangcheng Power Plant Shanxi. The content of SiO_2_ is between 40% and 50%, and it is the main component of the vitreous and the main substance that forms the hydro gel of calcium silicate. With the increase of SiO_2_, the activity of fly ash also increases, but the activity needs to be stimulated.

#### Lime and compound activator

Lime can be used for alkaline excitation of fly ash. The reason is hydrated cementitious products required by Ca^2+^ can be provided by lime, at the same time, the strong alkalinity of lime can erode the glass shell of fly ash and cause it to break, so that the activity of the fly ash can be stimulated. It can react with alkaline oxides (SiO_2_ and Al_2_O_3_ et al) and produce calcium silicate hydrate and calcium aluminates hydrate (as shown in Eqs 17 and 18). When fly ash and lime are mixed, the hydration rate is slow at normal temperature. Therefore, in order to improve the degree of reaction and accelerate the formation of hydrated calcium silicate and calcium aluminates hydrate, compound activator is researched. The compound activator is mainly composed of activator, early strength agent and expansion agent.


$${\mathrm{SiO}}_{2}+x{\mathrm{Ca(OH)}}_{2}+(n‐1)H_{2}O=x\mathrm{CaO}\cdot {\mathrm{SiO}}_{2}\cdot nH_{2}O$$ (17)

$${\mathrm{Al}}_{2}O_{3}+x{\mathrm{Ca(OH)}}_{2}+(n‐1)H_{2}O=x\mathrm{CaO}\cdot {\mathrm{Al}}_{2}O_{3}\cdot nH_{2}O$$ (18)

#### Experimental study on material proportioning

The fluidity, expansion rate and uniaxial compressive strength of the filling material are determined by fly ash, lime and compound activator.

The fluidity performance and uniaxial compressive strength for filling material are shown in Table 1. The experimental results indicated that the influence of fly ash and lime on the fluidity was not obvious. With the increase fly ash and lime proportion, the fluidity increases, that is, the fluidity of group a (fly ash: lime is 75:20), b (fly ash: lime is 80:15) and c (fly ash: lime is 85:10) are 225 mm, 238 mm and 245 mm, respectively. Due to the fly ash bleeding performance, certain expansion rate is required for the filling material to balance out the bleeding shrinkage. The most ideal experimental expansion rate (24h) of group b is 1.8%. The compressive strength of group a, b and c were 5.54, 6.47 and 4.52 MPa, respectively. By contrast, the compressive strength for group b specimens was improved by 117 and 143% compared to group a and c with a 90 days curing.

**Table 1:** Performance parameters of filling materials.

No.	Fly ash /%	Lime /%	compound activator B /%	Fluidity /mm	Expansion rate/%	Compressive strength /MPa		
						7d	28d	90d
**a**	75	20	5	225	0.45	0.65	2.79	5.54
**b**	80	15	5	238	1.8	0.73	3.32	6.47
**c**	85	10	5	245	1.1	0.50	2.25	4.52

The main reason is that the major active substance in fly ash is vitreous which are covered by glass shell formed under high temperature. This glass shell hindered the activity of fly ash and the lime effect takes time to develop which resulted in a lower strength in early stage. More lime had been added in group a which led to an intensive hydration reaction in the slurry after hardening and caused tiny cracks. The strength of group c was only 2.25 MPa. The main reason is the low lime content caused a weak alkaline environment in the slurry which limited the activity of fly ash. Group b has the best strengths, which are 3.32 and 6 MPa with 28 and 90 days curing respectively. Therefore, the best ratio of fly ash: lime was determined as 80:15.

**Fig 3:**
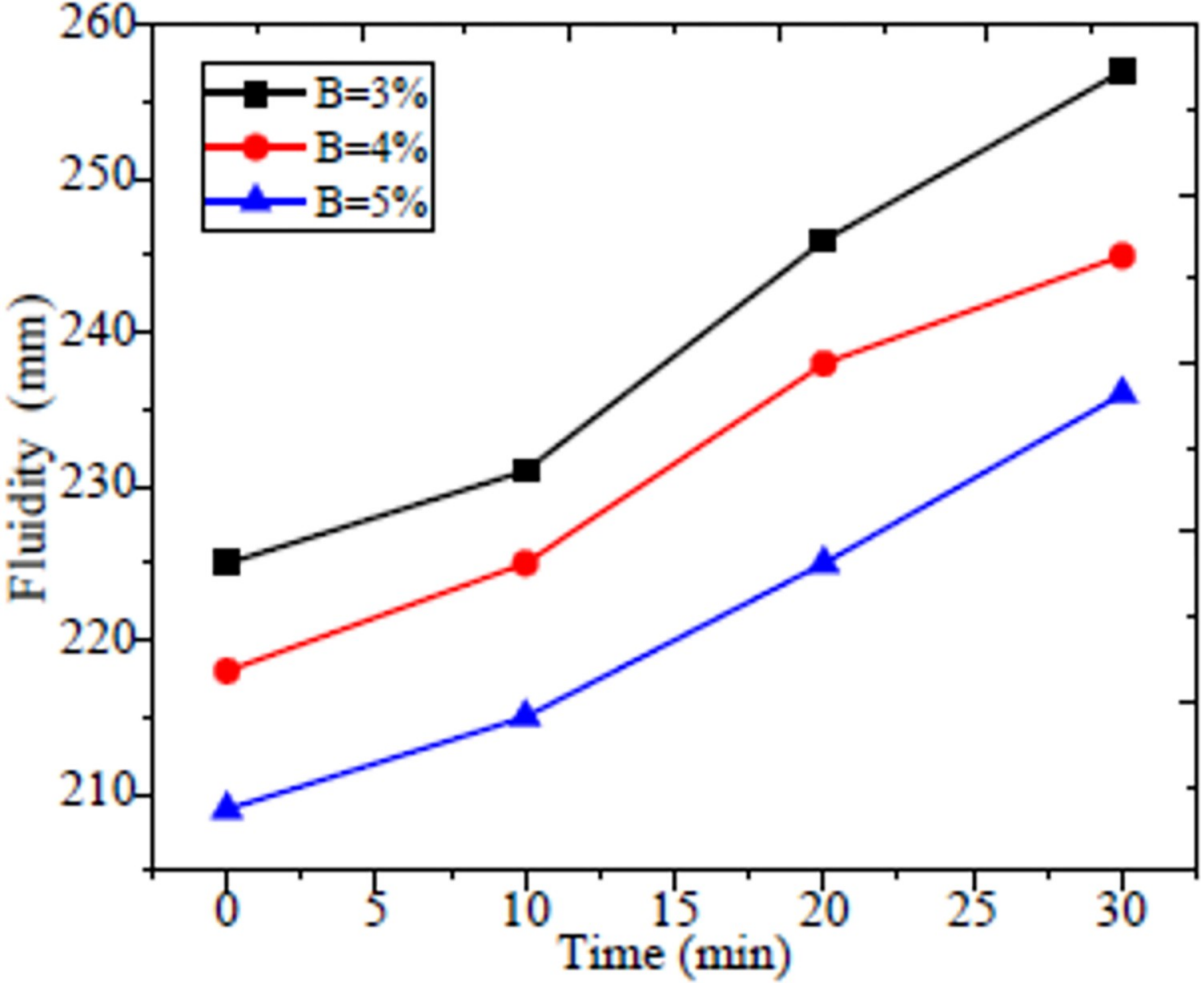
Fluidity of filling material.

**Fig 4:**
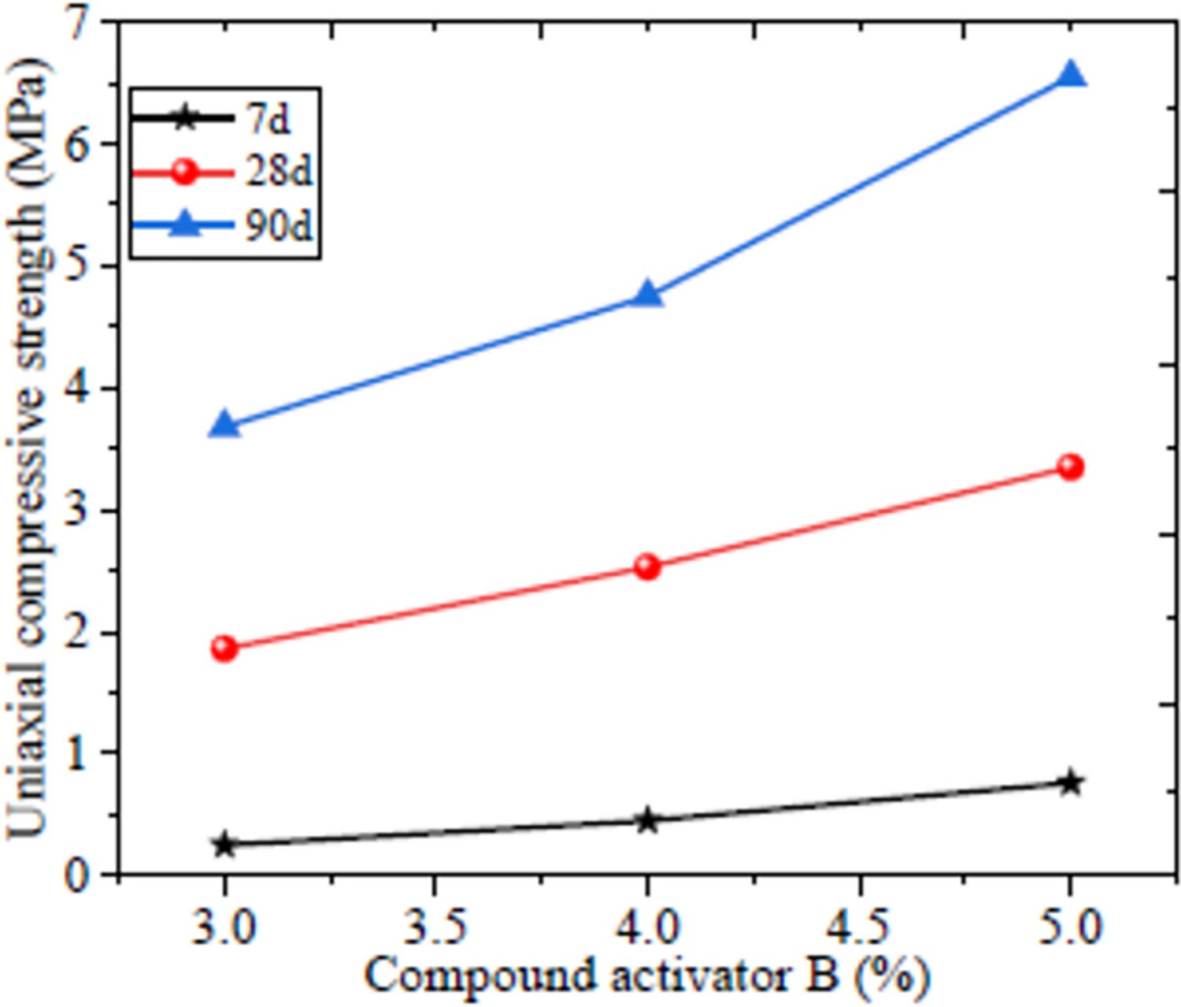
Expansion rate of filling material.

The compound activator B can improve the slurry fluidity, expansion rate, compressive strength and slurry bleeding contraction, hence it plays an important role in the slurry transportation. The influence of different proportions on the properties of the filling material was shown in Fig 3. It can be seen that with an increase content of compound activator B, the fluidity decreases. Specifically, the fluidity dropped to 257, 245 and 236 mm when the compound activator B increased from 3 to 4 and 5% respectively with a 30 min measure time. With the increase of compound activator B, the 24 h expansion rate of filling body was obviously improved as shown in Fig 4. When the content of compound activator B is 3%, its 3% expansion rate cannot balance out the low connection rate which was caused by bleeding shrinkage. When B content increased to 4 and 5%, the expansion rate reached to 1.3 and 2.4%, and the expansion rate was improved by 4.3 and 8.0 times compared to 3% compound activator B enhanced filling material.

**Fig 5:**
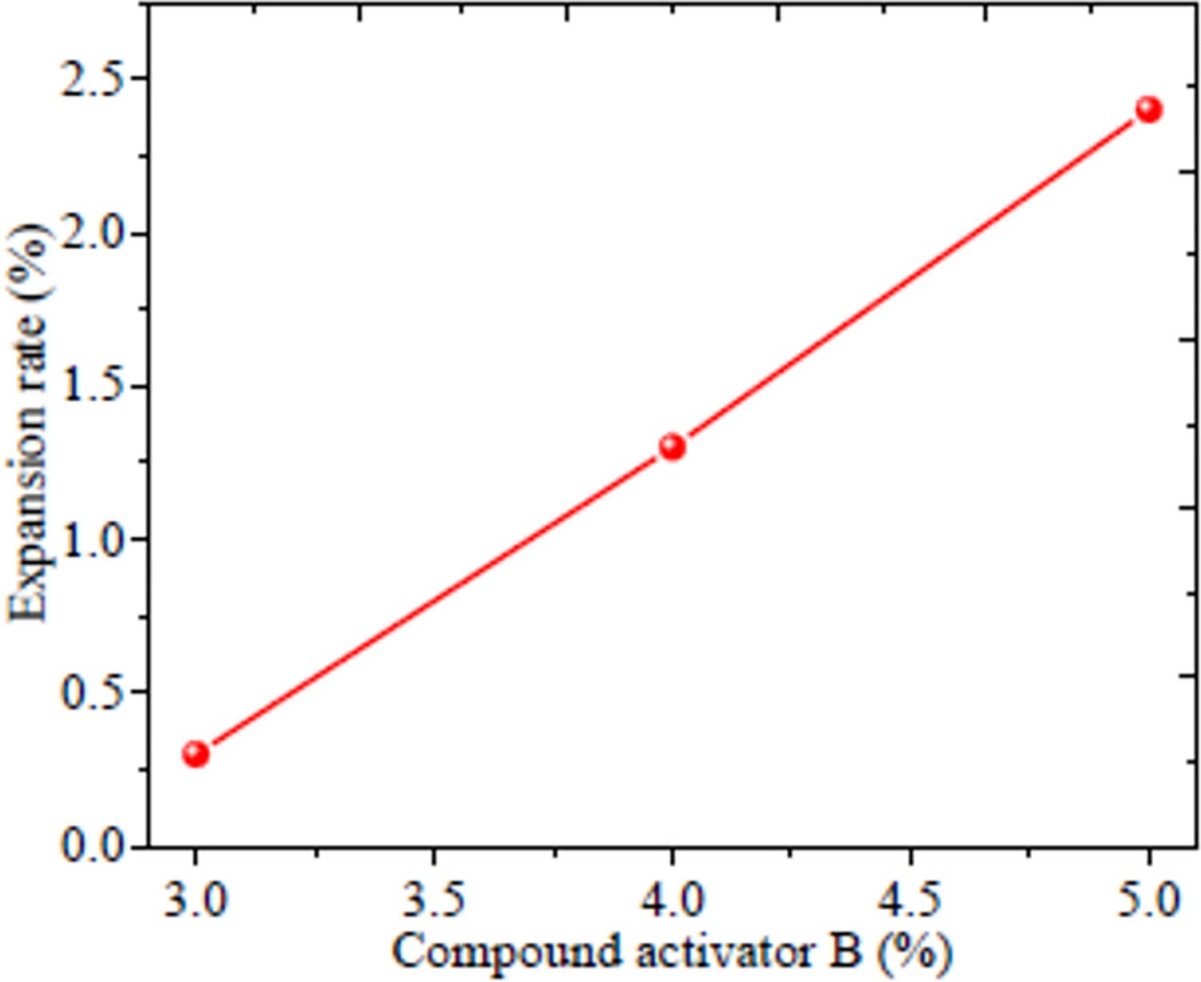
Compressive strength of filling material.

The compressive strengths with 90 days curing were 3.68, 4.75 and 6.55 MPa correspond to 3, 4 and 5% compound activator B content. It is clear that the compressive strength with 4 and 5% compound activator was improved by 29.1 and 77.9% compared to 3% compound activator as shown in Fig 5. The main reason is the increased compound activator B further eroded the glass shell of fly ash based system, meanwhile it also promoted the hydration reaction and promptly increased the compressive strength. Based on the theoretical calculation of the strength and the experimental analysis, the compound activator B was determined to be 5%.

#### Influence of water-cement ratio on filling material

After the material composition ratios were determined, the experimental water cement ratio (W/C) was set as 0.6:1, 0.7:1, 0.8:1, 0.9:1, 1.0:1. At 0.6 W/C, the slurry was stirred for 30 min, the fluidity of the slurry was only 200 mm, which cannot guarantee the fluidity of the slurry and even led to pipe blockage. When the W/C increased to 0.7, the initial fluidity of slurry was 218 mm, then the fluidity was 245 mm after stirring for 30 min with an expansion rate of 2.5%, and the shrinkage phenomenon did not occur. Meanwhile, the 28 and 90 days cured compressive strengths 2 reached to 3.35 and 6.62 MPa respectively. At the same time, the performance of the filling material had good workability and supporting strength, which not only supported a smooth pipe flow, but also had the characteristics of high roof closing rate. When the W/C increased to 0.8, the slurry fluidity was good, but a slight bleeding phenomenon was observed, and the 90 days cured compressive strength was 3.98 MPa. When the water cement ratio continued to increase, the flow rate was increased and the intensive bleeding shrinkage phenomenon was observed, and the strength decreased significantly. Considering the effective supporting capacity of the roof, the most ideal water cement ratio W/C = 0.7:1 was determined.

### Discussion on the optimal ratio performance

#### Viscosity time varying characteristics

**Fig 6:**
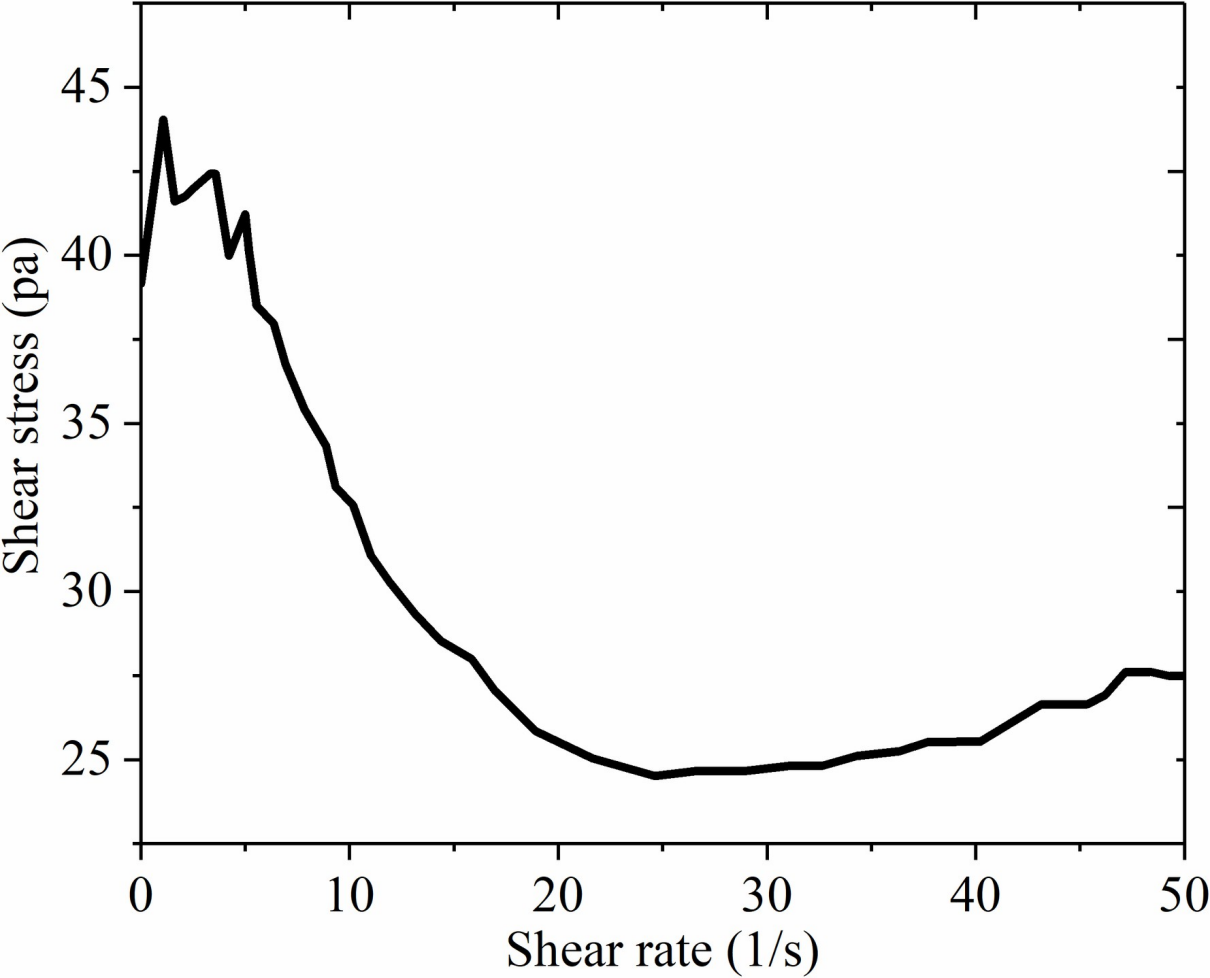
Initial 2min.

In order to verify that filling performance of the filling body, the viscosity, deformation characteristics and solubility are further confirmed. The change of the viscosity plays an important role in the slurry transportation and pipeline selection: therefore, the viscosity of the filling material was studied. The viscosity curve was measured by the rotating rheometer and the test results are shown in Fig 6.

**Fig 7:**
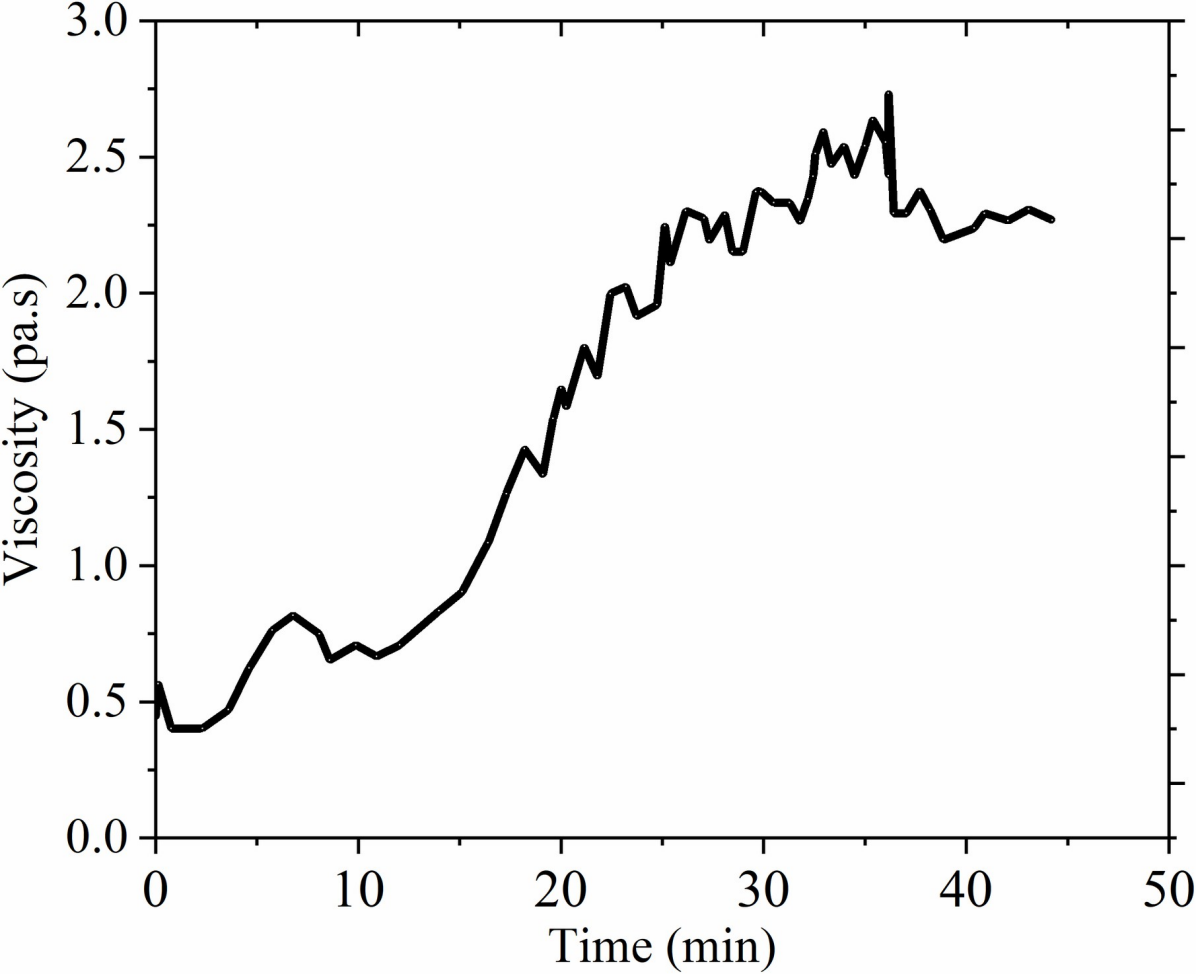
Viscosity curve.

Fig 6 showed the rheological cure when the serous reaction last for 2 min. Because the rheological index was negative, it conformed the slurry is pseudo plastic fluid. The concluded flow characteristics are the apparent viscosity decreased with the increase of shear rate, and the fluidity increased with the increase of shear rate, meanwhile, it presented the fluid thinning phenomenon was observed. The main reason is that due to the physical and chemical reaction among particles, the loose structure was formed. In addition, with the increase of shear strain rate, the structure was gradually destroyed, and the long chain molecules were rearranged along with the flow direction, which reduced flow resistance and apparent viscosity. As seen in Fig 7, flow time accumulation led to the increase of flow resistance and apparent viscosity. Therefore, it was concluded that the fly ash material presented the pseudo plastic fluid characteristics in the early filling stage, and it showed the characteristics of dilatant fluid in the later stage. By analyzing the time-varying viscosity characteristics of filling material, it provided guidance for the selection of slurry filling time and the filling pipeline design.

#### Discussion on water solubility of filling material

**Fig 8:**
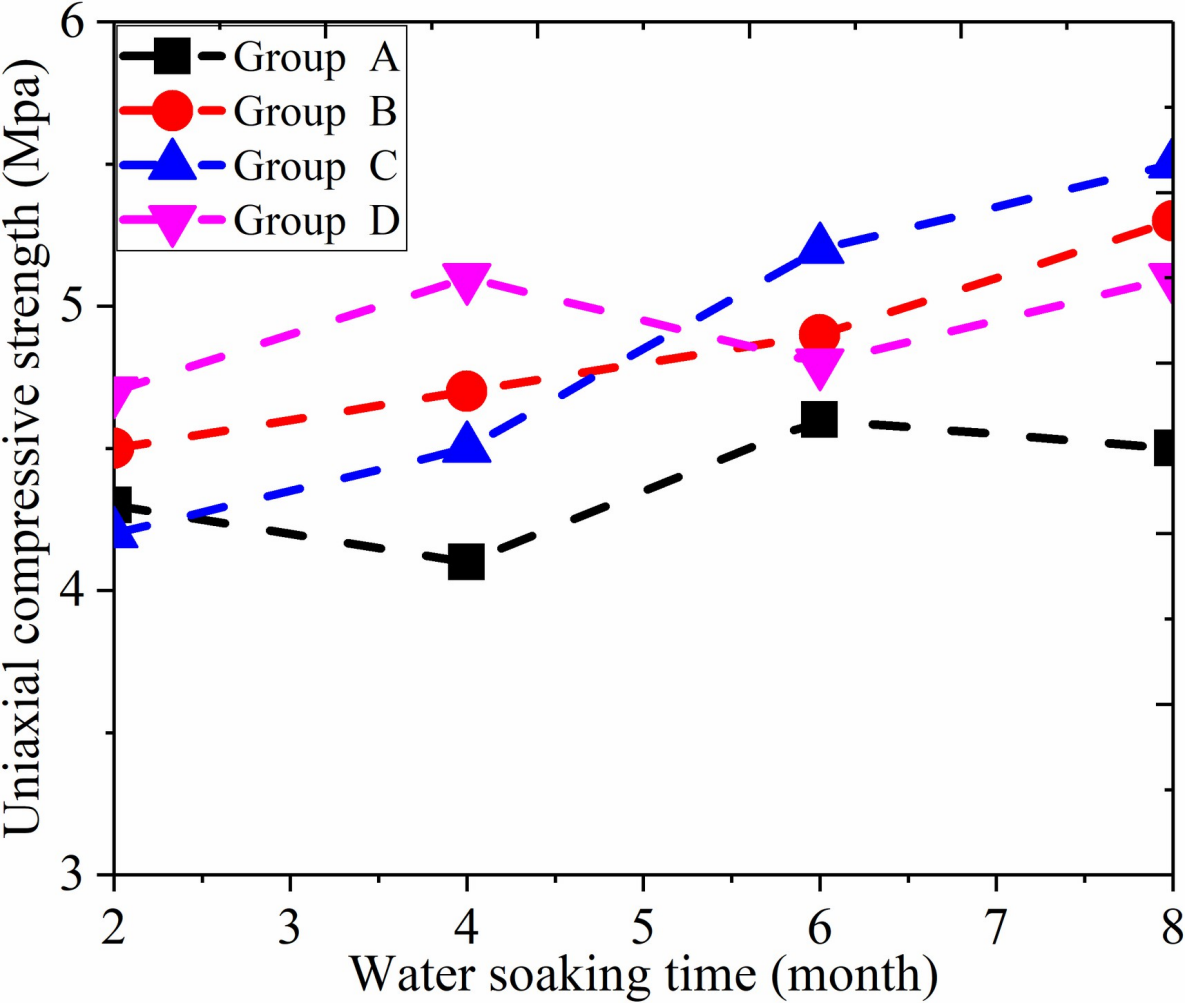
Compressive strength of filling material under immersion condition.

Groundwater can affect the performance of filling body and reduce compressive strength. Therefore, four group experiments (A, B, C and D) of water solubility were carried out. The initial strength of the filling body was set as two months, and the uniaxial compressive strength was tested after soaking for four months, six months and eight months. Test results shown in Fig 8 illustrated that the soaking time has no obvious impact on the initial uniaxial compressive strength, as the initial uniaxial compressive strength for group A, B, C and D were 4.3, 4.5, 4.4 and 4.5 MPa respectively. After soaking for six months, the uniaxial compressive strength increased a little to 4.5, 4.8, 4.7 and 4.8 MPa respectively. The results showed that the uniaxial compressive strength with a six months soaking were increased by 4.6, 6.6, 6.7 and 4.4% respectively. It indicated that the water solubility of the filling body was good, the uniaxial compressive strength was not affected by the immersion time, and the strength had an increasing trend.

In order to further simulate the confining pressure in coal mining, the triaxial compression experiment was conducted. The results showed that the strength of filling body increased obviously with the increase of confining pressure. And the compressive strength was more than 12 MPa under the confining pressure of 1.5 MPa, which is improved by 260% compared to its uniaxial compressive strengths. Meanwhile, relatively large plastic deformation and compression resistance were obtained. In this way, the filling body can maintain in the confining pressure state, the bearing capacity can be increased and the plastic yielding can be prevented.

#### Discussion on the filling effect

In order to ensure the stability and safety of reclaimed working face, the filling technology was researched, the ground and underground combined filling system was established. The filling effect was further verified.

#### Test of filling body reaction temperature

**Fig 9:**
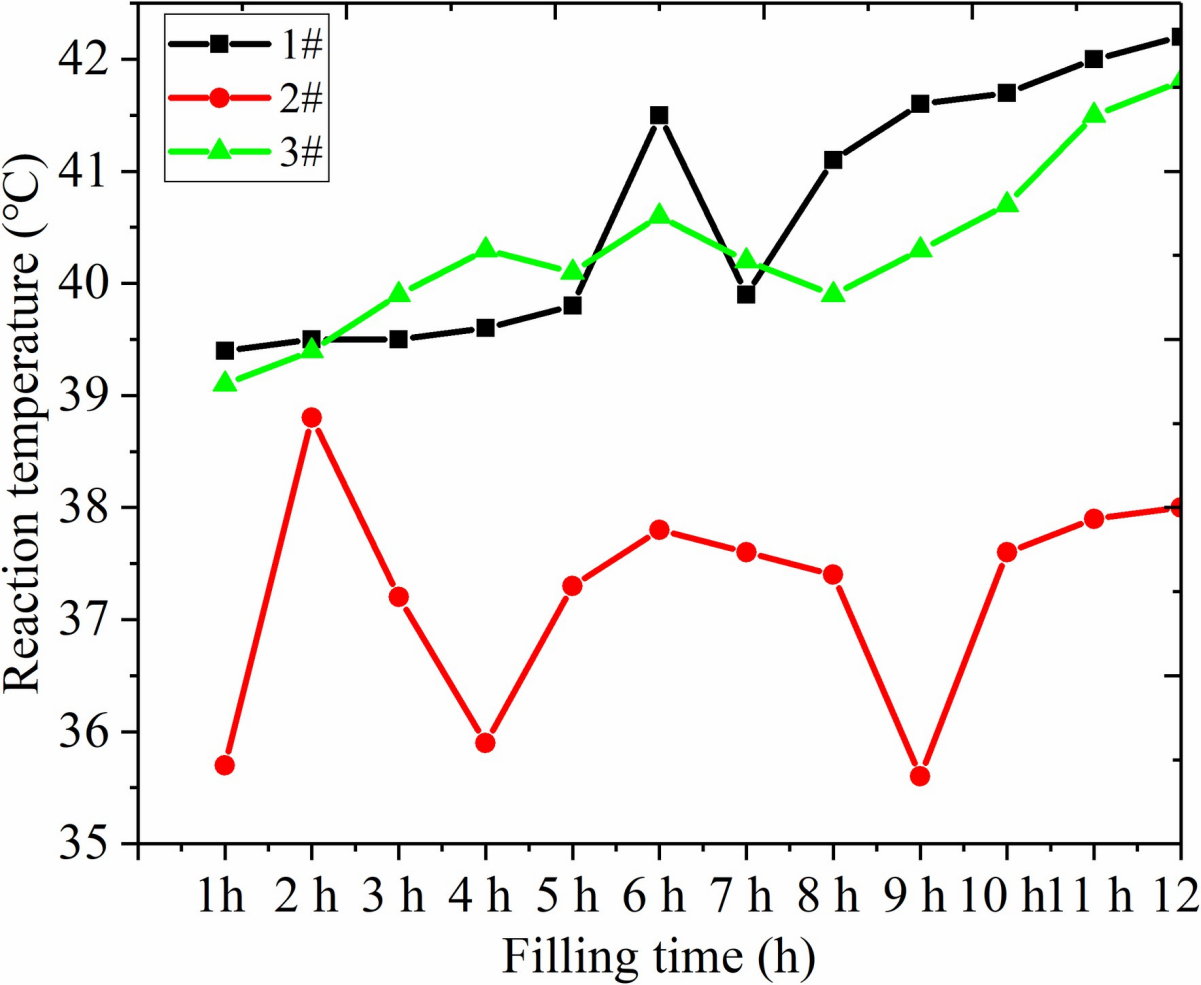
Temperature monitoring chart of underground filling body.

The filling material undergoes a strong hydration reaction during the hydration process, which releases a large amount of heat, leading to an increase in temperature. Due to the complex environment of coal mines, high slurry reaction temperatures can cause various safety issues. Therefore, three filler sensors were used to study the reaction temperature of the slurry. Measure the filling body temperature every 1 hour, as shown in Fig 9.

From the Fig 9, it was clear that the temperature of filling body in abandoned roadway gradually increased over time, but the increment was not obvious which is always around 40°C. The initial temperature for three groups were 39.4, 35.7 and 39.1°C, respectively, and the temperature increased to 42.2, 38.0 and 41.8°C, and then temperatures at 12 h increased to 7.1, 6.4 and 6.9% respectively. Therefore, the temperature caused by hydration reaction was relatively stable, which can meet the safety requirements of the underground mining. In addition, 40°C can promote the hydration reaction and accelerate the production of hydration products.

#### Performance test of filling body

The bleeding rate, expansion rate and compressive strength were tested for the investigation of filling body performance. First, five test points were selected for bleeding experiment, then the expansion rate test was carried out, and the uniaxial compressive strengths was measured by coring method. The results showed that the initial slurry bleeding rate remained at 5%, then the water completely disappeared and obtained initial strength after 12h, the average bleeding rate was 2.5%, which was close to the laboratory test. The uniaxial compressive strengths with 28 and 90 days curing were 3.5 MPa and 6.0 MPa respectively. When working face passed through the abandoned roadway, the surface of the filling body was intact without obvious coal wall caving and breakage. It proved that the filling body had good bond performance and filling effect.

#### Mine pressure monitoring at working face

**Fig 10:**
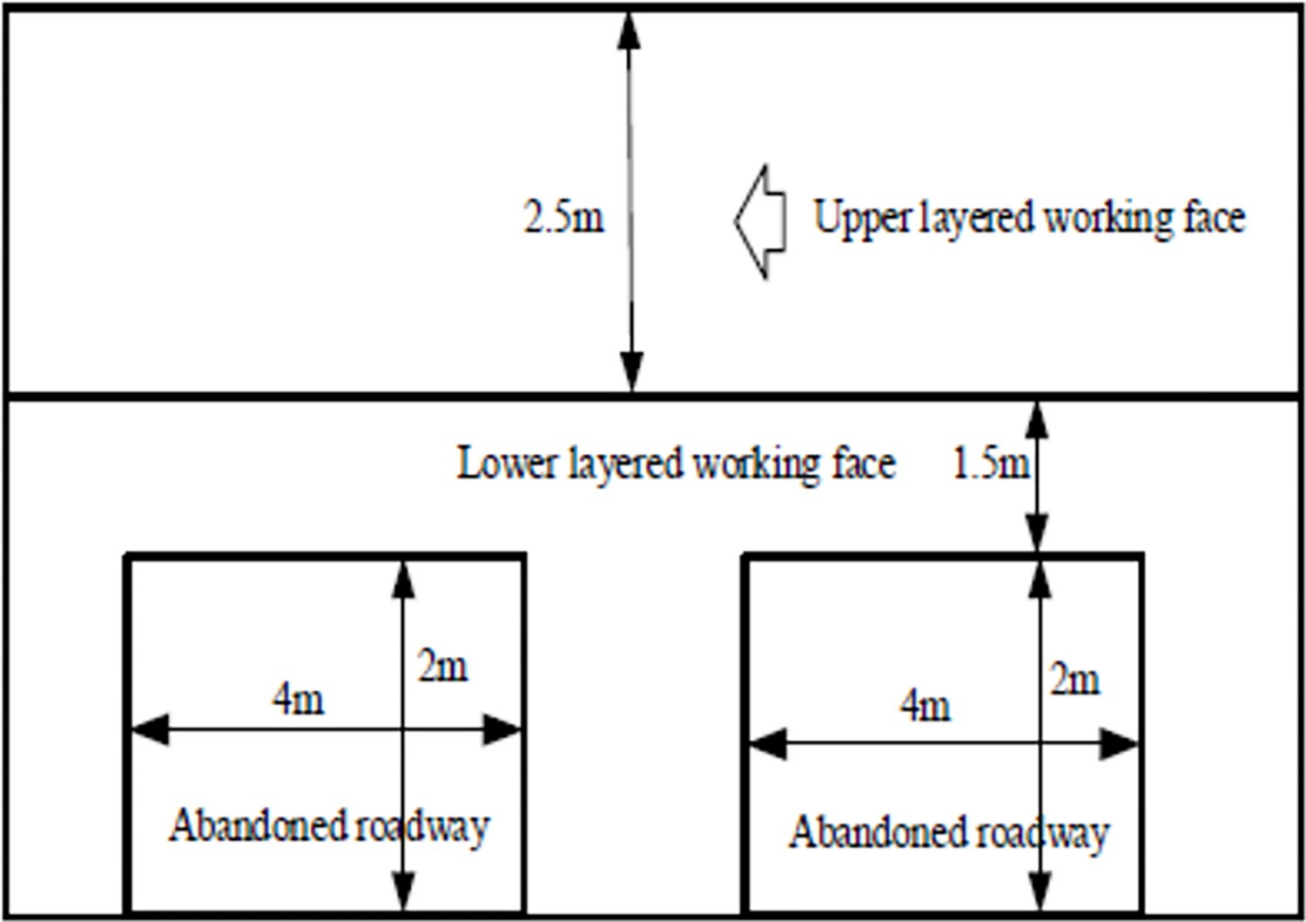
The working face and abandoned roadway profile.

The prime objectives of the field investigation were solving the roof stability issues under the advancing influence when the reclaimed working face passes through abandoned roadway. Hence, the field test was carried out on the working face in Shanxi. The mining height of upper working face is 2.5 m, and the lower working face is 3.5 m. Firstly, when the working face was mined, the working face passed through two abandoned roadways, and the parameters are shown in Fig 10.

In order to understand the behavior of mining induced stress in working face, 3 measuring lines in front, middle and rear were designed. Mine pressure collectors were installed at each measuring point on the working face, and mining induced stress behavior was analyzed when the working face passed through abandoned roadway. The data was recorded every 10s, and the online monitoring analysis was implemented when the working face was advancing to the abandoned roadway.

**Fig 11:**
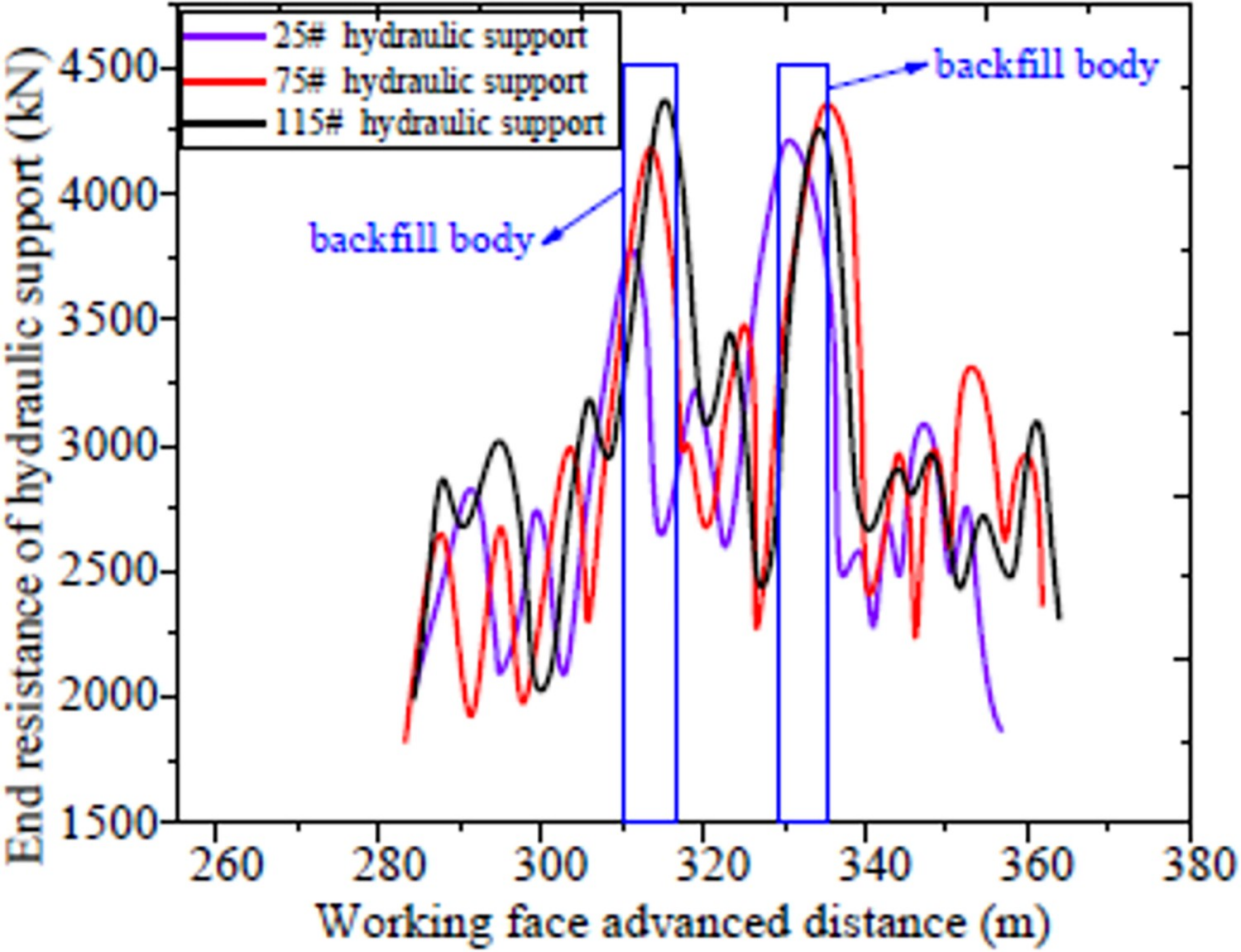
Load change diagram of hydraulic support.

Fig 11 revealed that the roof pressure of the reclaim working face was similar to that of the normal working face during the mining process. However, the roof pressure increased obviously when the working face was advancing to the filling lane. The main reason is that the filling strength is lower than that of the normal coal seam. When the working face advanced to the abandoned roadway, the floor of the abandoned roadway suddenly became soft which caused the settlement of the hydraulic support near the filling alley. Meanwhile, the roof also caves in with the hydraulic support, which led to the accumulation of roof in-situ pressure. It is because the filling material has large bearing capacity and compression resistance, in addition, the width of the alley is only 4 m with a slope. Therefore, the measured subjected pressure on the hydraulic support is within the range of its capacity. As shown in Fig 8, when the reclaim working face crossed the abandoned roadway, the maximum pressures of 25#、75#、and 115# hydraulic supports were 4482、4427 and 4317 kN, respectively. The above analysis showed that this filling technology can secure the safe advancement of the reclaim working face.

## Conclusions

In this article, the physical and mechanical properties of filling material were investigated. Meanwhile, the performance of the filling material was verified through the field application, and the conclusions can be obtained as follows:

1. The mechanical model of main roof in abandoned roadway was established, the stability in abandoned roadway was analyzed thematically, and the final supporting strength was determined as 3.3 MPa.
2. The filling material test results show that the average expansion rate of the filling material is 2.5%, and the compact filling rate is almost 100%. The uniaxial compressive strength after 28 and 90 days of curing is 3.5 and 6.0 MPa, respectively. The best ratio of fly ash, lime and composite activator B is 80:15:5, and the ideal Water-cement ratio is 0.7:1.
3. The experiment shows that the fly ash material exhibits pseudo plastic fluid characteristics during the initial filling stage. The compressive strength was more than 12 MPa under the confining pressure of 1.5 MPa. Obvious plastic deformation and compression resistance under the triaxial loading experiment were observed. The uniaxial compressive strength was not affected much by soaking in water, and the water solubility was better.
4. Field test proved that the hydration temperature of filling body was relatively stable and basically maintained at 40°C. The measured pressure on the hydraulic support is within its load-bearing capacity range, indicating that the filling material has good load-bearing capacity. And the filling body surface was quite complete. no obvious roof falling phenomenon occurred when working face passed through the abandoned roadway.

## Supporting information

S1 File (XLSX)
